# Current status and considerations on biosimilar in China

**DOI:** 10.1186/1479-5876-10-S2-A38

**Published:** 2012-10-17

**Authors:** Junzhi Wang

**Affiliations:** 1 National Institutes for Food and Drug Control, NIFDC, Beijing 100050, China

## 

Since the WHO “Guidelines on Evaluation of Similar Biotherapeutic Products (SBPs)” was published in April 2010[1], twenty two Countries and regions including EU and FDA have established their guidelines or guideline drafts on evaluation of biosimilar.

In terms of the technical features, WHO guidelines are consistent with that of EU[2-4], head-to-head comparison exercise between SBP and reference biotherapeutic product (RBP) are required. In the prerequisite of quality similarity, the unnecessary or repeated non-clinical and/or clinical data might be reduced. Furthermore, extrapolation to other indications of the RBP may be possible. As for the FDA draft, requirements of interchangeability and substitution of SBP with RBP were introduced [5,6]. Guidelines of other developing countries generally used the WHO guidelines and the FDA guidance draft as blueprints to set up the technical requirements. These regulations and guidelines have far-reaching significance for each country’s biotechnology industry development and the availability and affordability of public medicine use.

Up to now, there are not yet specified regulations for SBPs in China. Based on “The Provisions for Drug Registration (SFDA Order 28)” [7], Part I “Biological Products for Therapeutic Use” of the appendix 3 includes fifteen categories, three of them (7, 10 and 15) are related to SBPs. With respect to the technical requirements, there are no essential differences between China and WHO guidelines. However, our current regulations pay more attention to the requirements for the new drug approval. Consequently, some candidate SBPs products which might be suitable for abbreviated licensure pathway still need to experience the complete non-clinical and clinical studies. In addition, extrapolating to other indications is not allowed in our current regulations.

Among the research projects of the "Twelfth Five-Year Plan" significant new drugs creation special, me too biothreapeutics are still the major part of the projects of biotechnology medicines. Therefore, accelerating the process of establishing our SBPs guidelines has great benefit for achieving the goal of ensuring the availability and affordability of public medicine and improving the development of our country’s biotechnology industry. Recently, our department has initiated the process of surveying the need to draft a specified SBP guideline. As some suggestions, due to very hard to obtain and very high costing of RBP would surely increase the difficulties of developing and evaluation of SBPs, how to define the requirement of RBP should be elaborately considered during the process of establishing our guidelines. Besides, special attention should be focused on how to perform the comparability exercise with RBP in the non-clinical and/or clinical study during the development of SBPs of therapeutic monoclonal antibodies. We believe that a SBPs guideline which considering both the actual situation of development of biomedicine in China and the general WHO framework would be established in the near future.

## References

[B1] GUIDELINES ON EVALUATION OF SIMILAR BIOTHERAPEUTIC PRODUCTS (SBPs) http://www.who.int/biologicals/areas/biological_therapeutics/BIOTHERAPEUTICS_FOR_WEB_22APRIL2010.pdf 10.1016/j.biologicals.2011.08.008 21907589

[B2] Committee for Medicinal Products for Human Use Guideline on similar biological medicinal products 2005 London, European Medicine Evaluation Agency (CHMP/437/04)

[B3] Committee for Medicinal Products for Human Use Guideline on similar biological medicinal products containing biotechnology-derived proteins as active substance: Quality issues 2006 London, European Medicine Evaluation Agency (CHMP/BMWP/49348)

[B4] Committee for Medicinal Products for Human Use Guideline on similar biological medicinal products containing biotechnology-derived proteins as active substance: nonclinical and clinical issues 2006 London, European Medicine Evaluation Agency (CHMP/BMWP/42832)

[B5] Scientific Considerations in Demonstrating Biosimilarity to a Reference Product http://www.fda.gov/downloads/Drugs/GuidanceComplianceRegulatoryInformation/Guidances/UCM291128.pdf

[B6] Quality Considerations in Demonstrating Biosimilarity to a Reference Protein Product http://www.fda.gov/downloads/Drugs/GuidanceComplianceRegulatoryInformation/Guidances/UCM291134.pdf

[B7] The Provisions for Drug Registration (SFDA Order 28) 2007 http://www.sfda.gov.cn/WS01/CL0053/24529.html

